# Fingertip coverage with uni-pedicled volar rotational advancement flap with large Z-plasty: a report on 112 cases

**DOI:** 10.1186/s13018-023-04047-2

**Published:** 2023-07-31

**Authors:** Seong Oh Park, Dae Kwan Kim, Hee Chang Ahn, Youn Hwan Kim

**Affiliations:** 1 Department of Plastic and Reconstructive Surgery, Hanyang University College of Medicine, 222 Wangsimniro, Seongdong-gu, Seoul, 04763 Korea; 2 Department of Plastic and Reconstructive Surgery, CHA University Bundang Medical Center, Seongnam-si, Gyeonggi-do Korea

**Keywords:** Advancement flap, Fingertip, Local flap, Neurovascular bundle, Z-plasty

## Abstract

**Background:**

Simple and safe fingertip reconstruction methods involve the use of local neurovascular islands flaps that can preserve functional length and sensitivity, and reconstruction with skin of the same texture. However, techniques involving flaps have numerous drawbacks and do not satisfy all the requirements for fingertip reconstruction. A particular problem is the persistence of contracture deformity due to lack of full flap advancement. We present a new technique using uni-pedicled volar rotational advancement flap with large Z-plasty, and describe the results of long-term follow-up.

**Methods:**

From October 1993 to December 2009, 112 fingers of 98 patients were covered with uni-pedicled volar rotational advancement flap with large Z-plasty after sustaining various types of injuries or finger pulp avulsion. A longitudinal incision was made along the lateral border of the digit and a large neurovascular volar flap was elevated just above the pulleys and flexor tendon sheath. To release tension, a large Z-plasty was applied at the metacarpophalangeal joint or interphalangeal joint crease. The final patient outcomes were reviewed retrospectively.

**Results:**

All fingertip injuries were treated without flap necrosis. Partial wound dehiscence was observed in two patients and average static two-point discrimination was 5.2 mm. There were no postoperative contracture deformities, joint stiffness, paresthesia, or hypersensitivity. Most patients were left with acceptable scarring and were free of postoperative pain and cold intolerance during the long-term follow-up.

**Conclusions:**

Our novel technique provides durable, completely sensate, and well-vascularized coverage of the fingertip with minimal discomfort to patients.

## Background

Fingertips are the terminal extensions of the hand and are the parts most frequently injured [1]. Fingertip injuries occur in patients of all ages, from infants in neonatal nurseries to nursing home residents [2]. Patient age, occupation, gender, and hand dominance as well as the cause of injury, associated medical problems, and anticipated future hand use must be considered in choosing the type of surgical treatment [2–4]. Successful repair of fingertip injuries requires knowledge of anatomy and reconstruction techniques, as well as sound surgical judgment. Options for managing fingertip injuries range from simpler methods to technically demanding ones [2–7].

Neurovascular island flap reconstruction is generally used to treat fingertip injuries because it can preserve sensation, is long-lasting and maintains finger length without bone shortening. Also, advancement techniques and modifications are simple and safe. However, previous advancement flaps have had numerous drawbacks and do not satisfy all the requirements of fingertip reconstruction; in particular, contracture deformities may persist due to a lack of full flap advancement [3–6]. In this article, we introduce our novel technique that prevents contracture and promotes primary donor closure with sufficient flap advancement. We also summarize the results of long-term follow-up.

## Methods

This investigation was approved by our Institutional Review Board. It was a retrospective study of 112 fingers (from 98 patients) that underwent coverage with uni-pedicled volar rotational advancement flap with large Z-plasty (Ahn’s volar flap) from October 1993 to December 2009. We applied this surgical method to the fingertip injury with amputee loss or severe crushing, that make replantation difficult. The type of finger (including thumbs) does not affect the indication for this surgery. Instead, fingertip defects up to two-thirds of distal phalanx were included. The static two-point discrimination (using the sensory nerve recovery grading modified by Mackinnon and Dellon), contracture deformities, digit stiffness, hypersensitivity, numbness, postoperative pain and cold intolerance were evaluated during the follow-up period and reviewed retrospectively.

### Surgical techniques

**Fig.1:**
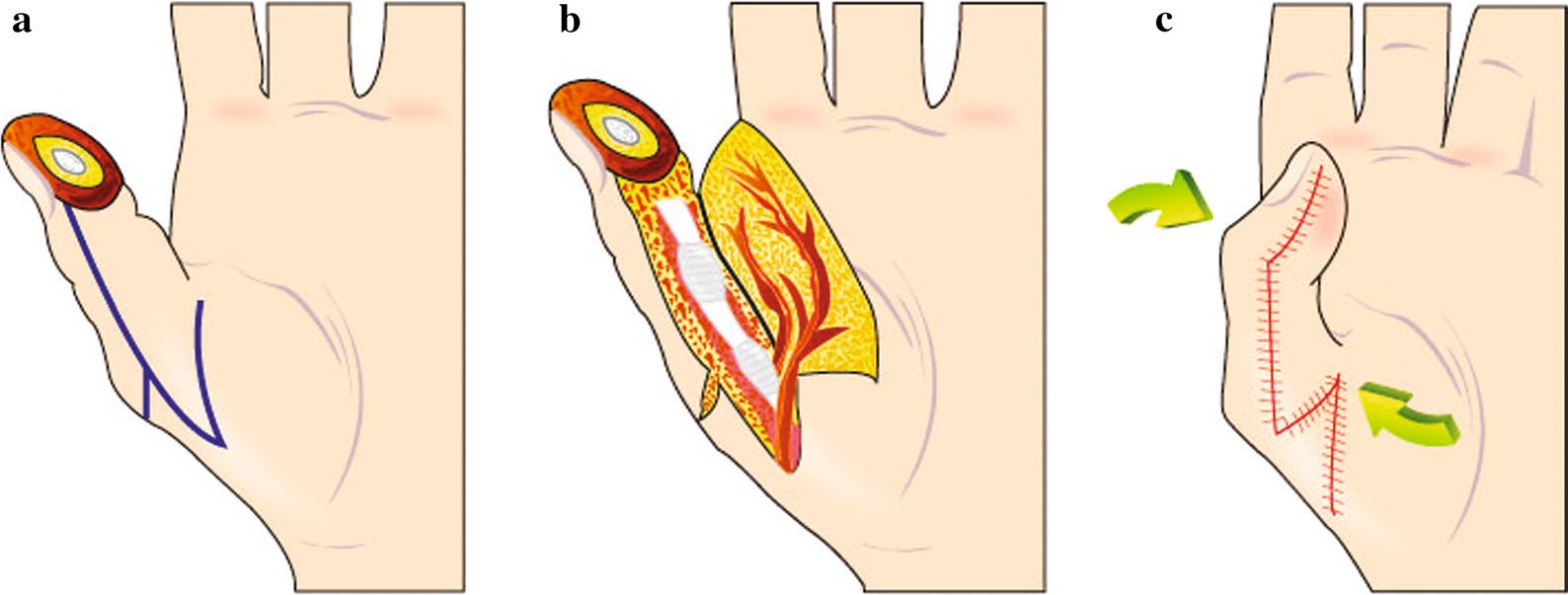
**a** A thumb injury and design of the flap. **b** A neurovascular flap was elevated just above the pulleys and sheath of the flexor tendon. **c**. The flap covered the thumb defect, and a large Z-plasty on the MP joint area released tension

Digital or arm tourniquet control and loupe magnification were used during flap elevation and dissection. A large neurovascular volar flap was elevated just above the pulleys and flexor tendon sheath after making a longitudinal incision along the lateral border of the digit. A large Z-plasty was applied at the meta-carpo-phalangeal (MCP) joint or interphalangeal (IP) joint crease to release tension. A mid-axial incision was made on either the radial or ulnar side of the finger from the amputation site to the MCP joint (Fig. 1a). Cleland’s ligaments were incised, and incisions were made across the dorsal side of the thumb to the neurovascular bundles, and on the superficial side of the thumb to the flexor tendon sheath. All the skin, subcutaneous tissue, and both neurovascular bundles were included in the flap (Fig. 1b). A large Z-plasty was used to lengthen the flap laterally on the MCP joint. The base of the flap was cut along the MCP joint crease, and the opposite extremity of the Z-plasty was created on the dorsum of the finger.

The distal corner of the flap was advanced and rotated to cover the tip of the finger, and sutured into its new location. This technique provides good coverage and eliminates the need for a graft. The IP joint was flexed and immobilized in a slight flexion for 10 days to allow for wound healing without tension (Fig. 1c). The same procedure can also be performed on other fingers in a similar fashion.

## Results

The age of the patients ranged from 3 to 64 years (mean, 32.4 years). There were 68 thumbs, 18 index fingers, 8 long fingers, 4 ring fingers, and 14 little fingers. The phalangeal bone was exposed by all the injures. The amputations were classified as transverse (*n* = 62), oblique palmar (*n* = 25), oblique radial (*n* = 12), or oblique ulnar (*n* = 13). The data are summarized in Tables 1 and 2.

**Table 1:** Location of amputation

Location	Number of digits	(%)
Thumb	68	60.7
Index finger	18	16.1
Long finger	8	7.1
Ring finger	4	3.6
Small finger	14	12.5
Total	112

**Table 2:** Type amputation

Type	Number of digits	(%)
Transverse	62	55.4
Oblique palmar	25	22.3
Oblique radial	12	10.7
Oblique ulnar	13	11.6

None of the patients had postoperative contracture deformities or reported joint stiffness; No postoperative infections, flap necroses, or hematomas were observed in any of the patients. However, two patients developed partial wound dehiscence, which was treated by secondary conservative management, and one patient suffered hypertrophic scarring at the linear incision site. This was mitigated by a series of steroid injections and compression.

The average static two-point discrimination was 5.2 mm (range, 4–8 mm). Using the sensory nerve recovery grading modified by Mackinnon and Dellon, there were 71 cases in the S4 grade, and 41 in the S3 + grade. The level of postoperative pain during long-term follow-up (at least 24 months) varied: 99 patients experienced no postoperative pain, 7 reported pain when tapping the injured finger, and another 6 complained of pain when the injured digit was at rest. Cold intolerance developed within 6 months in 10 cases, but it disappeared later in all but two cases (mean, 56 months). Two patients suffered paresthesia at the stump during the early postoperative period, which resolved during long-term follow-up. No hypersensitivity was noted. Table 3 summarizes the outcomes.

**Table 3:** Summary of outcomes

Mean age (year)	32.4
Mean follow up duration (month)	56
Mean static two-point discrimination (mm)	5.2

	Number of digits	%
Sensory nerve recovery grading^a^		
S0	0	
S1	0	
S2	0	
S3	0	
S3+	41	36.6
S4	71	63.4
Outcomes regarding operation		
Contracture deformities	0	
Digit stiffness	0	
Hypersensitivity	0	
Numbness	2^b^	
Postoperative pain		
No pain	99	88.4
Pain with tapping	7	6.3
Pain at rest	6	5.4
Cold intolerance	10^c^	
Complications related wounds	3^d^	

^a^Sensory nerve recovery grading modified by Mackinnon and Dellon (S0; Absence of sensibility in the autonomous zone of the nerve, S1; Recovery of deep cutaneous pain sensibility within the autonomous zone of the nerve, S2; Partial recovery of superficial pain and tactile sensibility within the autonomous area of the nerve, S3; Recovery of superficial cutaneous pain and tactile sensibility throughout the autonomous area without hyperalgesia (static two-point discrimination > 15 mm), S3+; Recover to S3 level and some recovery of static two-point discrimination 7-15 mm, S4; Complete recovery (static two-point discrimination 2–6 mm))

^b^Two patients suffered paresthesia early postoperatively, the symptom relieved during long term follow ups

^c^Cold intolerance developed in 10 cases in during the early follow-up periods (within 6 months). Fortunately, this disappeared in all cases except two during long-term follow-up

^d^Two patients suffered immediate wound dehiscence of closure site, it healed well conservatively. And the other one patient suffered hypertrophic scar which resolved by serial steroid injection with compression scar treatment

All the fingertip injuries were successfully covered without flap necrosis, and, with exercise, all the injured fingers rapidly regained a full range of motion. Most of the patients returned to normal activity approximately 6 to 8 weeks after their operations.

### Case 1

**Fig. 2:**
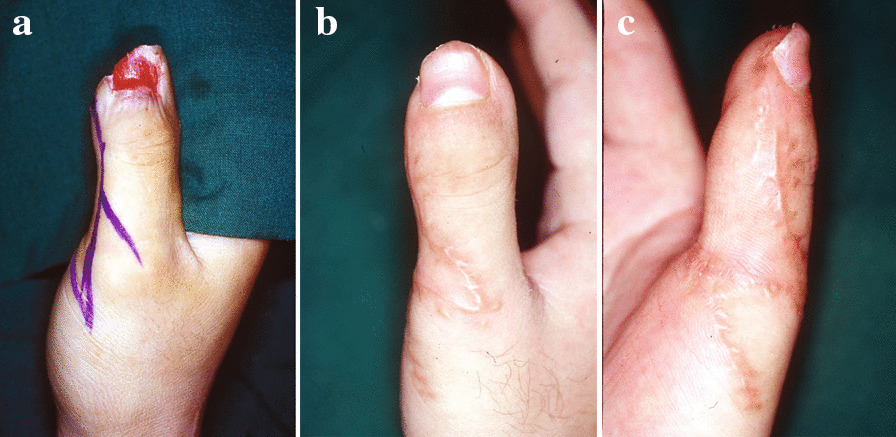
**a** Half of the radial side of the thumb pulp was avulsed with phalangeal bone exposure. **b**, **c** An identical flap was elevated using a standard procedure, and Z-plasty on the MCP joint crease released tension. A review after 3 years found that the fingertip had a good contour without any nail deformity. There was also no flexion contracture deformity of the thumb

In this 30-year-old man, half of the radial side of the thumb pulp was avulsed with small distal phalanx bone exposure. A flap identical to the one described in Fig. 1a was elevated by the standard technique. Z-plasty on the MCP joint crease served to release tension. Postoperatively, the fingertip maintained a good contour without nail deformity, and with no flexion contracture deformity of the thumb (Fig. 2).

### Case 2

**Fig. 3:**
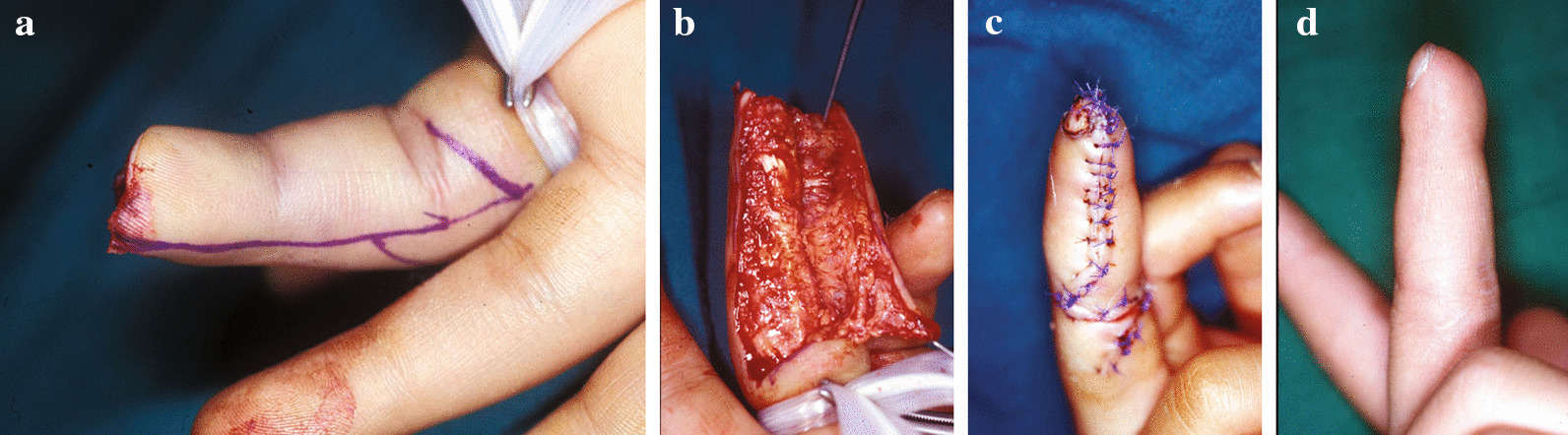
**a** The tip of the long finger had been amputated in an oblique direction. A midaxial incision was made on the radial side of the digit. **b** The flap was elevated using a standard technique. c. Immediate postoperative view showing primary **c**losure of the donor site without tension. **d** Wound healing was uneventful. Four years after the operation, the fingertip was well-covered by a sensate, durable flap. A new skin crease was made at the DIP joint

The tip of the long finger of this fourteen-year-old boy had been amputated in the oblique direction. A mid-axial incision was made on the radial side of the digit, and a flap was elevated using the standard technique. Wound healing was uneventful. Postoperatively (1 year), a sensate, durable flap maintained good coverage of the fingertip. A new skin crease formed at the distal interphalangeal (DIP) joint (Fig. 3).

## Discussion

The surgical goals of fingertip reconstruction should include the preservation of functional length and sensitivity, reconstruction using skin that has the same texture as the injury site, and prevention of symptomatic neuroma and contracture [2, 8–10]. To accomplish these goals, primary closure, secondary intention, and graft techniques are unsuitable. Flap reconstruction is the best way to satisfy all these requirements [2–14].

With recent technological advancements in wound healing, there has been research about using artificial dermis graft with stromal vascular fraction cell on fingertip defect. Although this method is simple and non-invasive, it may not be ideal for larger and deeper fingertip defects, and it could delay patient's return to daily activities due to a longer healing process compared to one-stage flap coverage [15].

Free flaps using microvascular transfer of a partial toe can satisfy all the requirements for fingertip reconstruction and has many advantages. However, it requires microsurgical techniques applied to small-sized vessels; hence, there is always a potential risk of vascular complications. Even if partial toe harvesting techniques are applied, there remain some concerns regarding donor digit sacrifice [2, 12].

Koshima et al. have developed a perforator technique using a digital artery perforator to treat fingertip injuries. This is a simple and representative method of fingertip reconstruction [16]. However, it does not include nerve structures and sensory recovery, which is of concern.

The use of a volar advancement flap has been advocated for sensate coverage based on the neurovascular island flap principle. This flap was first described by Littler [5] and Moberg [6], and various modifications have been described [17–20]. However, this flap is generally safe only for the thumb due to its good dorsal arterial blood supply. The blood supply of the other fingers to dorsal tissues distal to the proximal interphalangeal joint level is primarily delivered through branches from neurovascular bundles. Hence, elevation of these bi-pedicled patterns can impede vascularity of the distal fingertip [8, 10]. Scar contracture deformities can also form flexed contractures, which typically develop following Moberg’s volar advancement flap when treating thumb tip injuries, and linear incision along the neurovascular bundles often results in severe contracture [8, 10, 17–19].

Evans and Martin developed a modified advancement flap using a step-advancement flap to minimize scar contracture deformities by maximizing the advancement of volar flaps [9, 21]. As a result, the volar zigzag scars were inconspicuous. However, this type of step advancement flap still suffers from contracture because the incision line crosses the joint crease. In addition, it is very difficult to use to treat an injured thumb because the thumb is short compared to the other fingers, and increased advancement of the flap requires a long step pattern design, and is therefore not suitable for repairing thumb defects. This means that the linear advancement is not sufficient to cover large defects.

To overcome all the pitfalls of local advancement flaps, Hueston modified the volar advancement flap using a uni-pedicle technique [22] and performed rotation advancement of the volar thumb skin based only on the vascular pedicle of one side. However, the triangular secondary defect at the base of the thumb requires a full-thickness skin graft or triangular flap derived from the side of the thumb. The resulting scars are often unacceptable and can cause contracture deformities.

For primary closure of the donor site, Tuncali et al. modified the Hueston flap using a Hatchet-shaped design. This design originated from other reconstructive techniques, such as pressure sore reconstruction methods using back cut or distal Z-plasty techniques to release tension when the flap is rotated [8]. However, in our experience, scar contractures often develop in joint areas such as the sites of radial linear or ulnar linear incisions. Thus, volar Z-plasty, which was described by that group, can only be used for closure of the donor site rather than to prevent contracture deformities. Moreover, small Z-plasty cannot achieve tension release or flap advancement. On the other hand, in our method we use a uni-pedicled rotational advancement flap with large Z-plasty in the MCP or IP joints where contracture deformities often form in the absence of a graft. Moreover, we used this technique not only to treat injuries of the thumb but also those of other fingers [23].

Our Ahn’s volar flap (uni-pedicled volar rotational advancement flap with large Z-plasty) can prevent contracture deformity of the digits because it involves only one-sided mid-axial incisions and lengthening Z-plasty. In addition, tension applied in the oblique, but not longitudinal direction can prevent the beaked deformity associated with finger and flexion contracture [23]. Duration of wound healing has been reported to be an independent predictor of flexion contracture of the proximal interphalangeal (PIP) joint after application of a homodigital island flap [24]. That suggests that our method's large Z-plasty and one-sided mid-axial incision minimizes tension at the surgical site and reduces the time required for wound healing, and may have contributed to the absence of joint stiffness.

Ahn’s volar flap is obviously not superior to other methods in every situation where local tissue is used. However, it has many advantages, including good sensation and durability because neurovascular bundles are included. Average static two-point discrimination was measured to be 5.2 mm, which is comparable to other techniques. It also provides greater postoperative comfort and can be used to treat any type of fingertip injury in patients of all ages by means of a single-stage operation.

However, the limitation of this study is that we did not perform statistical comparisons with other representative methods of fingertip reconstruction. This was because our method is considered on empirical grounds to give better results than the other methods of volar defect reconstruction, with the result that the numbers of cases where these alternative methods have been used are insufficient for comparative purposes. Instead, we reviewed various representative methods of fingertip reconstruction, and considered the advantages and disadvantages of our method theoretically compared to each of the alternatives. Other limitation was the retrospective and uncontrolled design of the study.

## Conclusions

Based on the long-term results with our 112 cases, we believe that Ahn’s volar flap technique is a suitable option for reconstructing any type of finger at any location. The flap is durable, completely sensate, well vascularized, and can be used to treat fingertip injuries without further shortening of the bone. Moreover, the approach is easy and accessible without microsurgical techniques.

## Data Availability

The data that support the findings of this study are available from the corresponding author upon reasonable request.

## Abbreviations

Ahn’s volar flap: Uni-pedicled volar rotational advancement flap with large Z-plasty
MCP: Meta-carpo-phalangeal
IP: Interphalangeal
DIP: Distal interphalangeal
PIP: Proximal interphalangeal

## References

[1] Sorock GS, Lombardi DA, Hauser RB, Eisen EA, Herrick RF, Mittleman MA (2002). Acute traumatic occupational hand injuries: type, location, and severity. J Occup Environ Med.

[2] Lemmon JA, Janis JE, Rohrich RJ (2008). Soft-tissue injuries of the fingertip: methods of evaluation and treatment: an algorithmic approach. Plast Reconstr Surg.

[3] Atasoy E, Ioakimidis E, Kasdan ML, Kutz JE, Kleinert HE (1970). Reconstruction of the amputated finger tip with a triangular volar flap: a new surgical procedure. J Bone Joint Surg Am.

[4] Kutler W (1947). A new method for finger tip amputation. J Am Med Assoc.

[5] Littler JW (1953). The neurovascular pedicle method of digital transposition for reconstruction of the thumb. Plast Reconstr Surg.

[6] Moberg E (1964). Aspects of sensation in reconstructive surgery of the upper extremity. J Bone Joint Surg Am.

[7] Fassler PR (1996). Fingertip injuries: evaluation and treatment. J Am Acad Orthop Surg.

[8] Tuncali D, Barutcu AY, Gokrem S, Terzioglu A, Aslan G (2006). The hatchet flap for reconstruction of fingertip amputations. Plast Reconstr Surg.

[9] Hammouda AA, El-Khatib HA, Al-Hetmi T (2011). Extended step-advancement flap for avulsed amputated fingertip: a new technique to preserve finger length: case series. J Hand Surg Am.

[10] Foucher G, Dallaserra M, Tilquin B, Lenoble E, Sammut D (1994). The Hueston flap in reconstruction of fingertip skin loss: results in a series of 41 patients. J Hand Surg Am.

[11] Koshima I, Urushibara K, Fukuda N, Ohkochi M, Nagase T, Gonda K, Asato H, Yoshimura K (2006). Digital artery perforator flaps for fingertip reconstructions. Plast Reconstr Surg.

[12] Lee DC, Kim JS, Ki SH, Roh SY, Yang JW, Chung KC (2008). Partial second toe pulp free flap for fingertip reconstruction. Plast Reconstr Surg.

[13] Lai CS, Lin SD, Yang CC (1989). The reverse digital artery flap for fingertip reconstruction. Ann Plast Surg.

[14] Iwasawa M, Furuta S, Noguchi M, Hirose T (1992). Reconstruction of fingertip deformities of the thumb using a venous flap. Ann Plast Surg.

[15] Namgoong S, Jung JE, Han SK, Jeong SH, Dhong ES (2020). Potential of tissue-engineered and artificial dermis grafts for fingertip reconstruction. Plast Reconstr Surg.

[16] Güleç A, Özdemir A, Durgut F, Yildirim A, Acar MA (2019). Comparison of innervated digital artery perforator flap versus homodigital reverse flow flap techniques for fingertip reconstruction. J Hand Surg Am.

[17] Keim HA, Grantham SA (1969). Volar-flap advancement for thumb and finger-tip injuries. Clin Orthop Relat Res.

[18] Posner MA, Smith RJ (1971). The advancement pedicle flap for thumb injuries. J Bone Joint Surg Am.

[19] Macht SD, Watson HK (1980). The Moberg volar advancement flap for digital reconstruction. J Hand Surg Am.

[20] Feng SM, Zhao JJ, Migliorini F, Maffulli N, Xu W (2021). First dorsal metacarpal artery flap with dorsal digital nerve with or without dorsal branch of the proper digital nerve produces comparable short-term sensory outcomes. J Orthop Surg Res.

[21] Evans DM, Martin DL (1988). Step-advancement island flap for fingertip reconstruction. Br J Plast Surg.

[22] Hueston J (1966). Local flap repair of fingertip injuries. Plast Reconstr Surg.

[23] Ahn HC, Ahn DK (1997). Fingertip coverage with large neurovascular rotation flap and Z-Plasty. J Korean Soc Plast Reconstr Surg.

[24] Nakanishi A, Omokawa S, Iida A, Kaji D, Tanaka Y (2015). Predictors of proximal interphalangeal joint flexion contracture after homodigital Island flap. J Hand Surg Am.

